# Marker-Based Method for Recognition of Camera Position for Mobile Robots

**DOI:** 10.3390/s21041077

**Published:** 2021-02-04

**Authors:** Dong-Gi Gwak, Kyon-Mo Yang, Min-Ro Park, Jehun Hahm, Jaewan Koo, Joonwoo Lee, Kap-Ho Seo

**Affiliations:** 1 HRI Research Center, Korea Institute of Robotics & Technology Convergence, Pohang 37553, Korea; dg.gwak@kiro.re.kr (D.-G.G.); kmyang@kiro.re.kr (K.-M.Y.); minro@kiro.re.kr (M.-R.P.); jhham@kiro.re.kr (J.H.); jwkoo3236@kiro.re.kr (J.K.); 2 Department of Robot and Smart System Engineering, Kyungpook National University, Daegu 41566, Korea; jwl@knu.ac.kr; 3 Department of Electrical Engineering, Kyungpook National University, Daegu 41566, Korea; 4 School of Electronic and Electrical Engineering, Kyungpook National University, Daegu 41566, Korea; 5 Department of Mechanical Engineering, Pohang University of Science and Technology, Pohang 37673, Korea

**Keywords:** artificial marker, indoor localization, mobile robot, position recognition

## Abstract

Position recognition is one of the core technologies for driving a robot because of differences in environment and rapidly changing situations. This study proposes a strategy for estimating the position of a camera mounted on a mobile robot. The proposed strategy comprises three methods. The first is to directly acquire information (e.g., identification (ID), marker size and marker type) to recognize the position of the camera relative to the marker. The advantage of this marker system is that a combination of markers of different sizes or having different information may be used without having to update the internal parameters of the robot system even if the user frequently changes or adds to the marker’s identification information. In the second, two novel markers are proposed to consider the real environment in which real robots are applied: a nested marker and a hierarchical marker. These markers are proposed to improve the ability of the camera to recognize markers while the camera is moving on the mobile robot. The nested marker is effective for robots like drones, which land and take off vertically with respect to the ground. The hierarchical marker is suitable for robots that move horizontally with respect to the ground such as wheeled mobile robots. The third method is the calculation of the position of an added or moved marker based on a reference marker. This method automatically updates the positions of markers after considering the change in the driving area of the mobile robot. Finally, the proposed methods were validated through experiments.

## 1. Introduction

**Figure 1:**
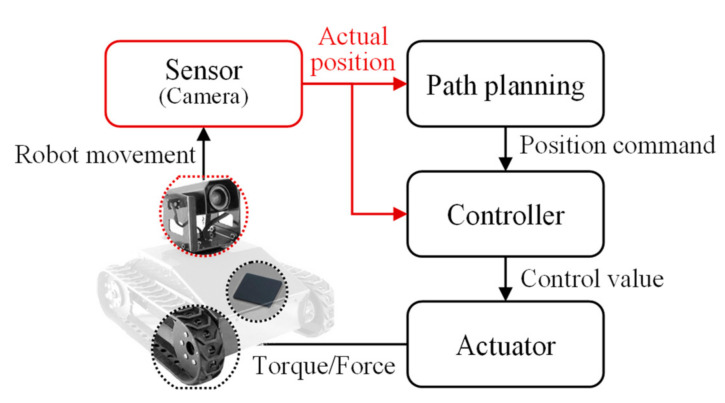
Driving mechanism of the mobile robot.

Mobile robots are widely used in various fields such as entertainment, medicine, rescue, the military, space, and agriculture [1]. Therefore, the technological development and performance improvement of mobile robots have been investigated in numerous aspects. In particular, studies are being actively conducted on the path planning of mobile robots in a given environment and on the control required to accurately move a robot along a planned path [2,3,4,5,6,7,8]. Figure 1 shows a simple driving mechanism for a mobile robot. A path is appropriately planned based on the surrounding environment. The location of the mobile robot must be accurately recognized to correctly perform a given command. Its position and direction information is identified through position recognition, the core technology of mobile robots [9] Position recognition, broadly speaking, can be either outdoor or indoor, where the former is mainly studied using the global positioning system (GPS) [10]. However, it is difficult to apply the GPS to indoor position recognition because it requires precise location information [11], so vision-based position recognition technology is used. This technology applies to position recognition based on the features of the environment and recognition of an artificial marker-based position [12]. Position recognition that uses features does not require additional work to be applied to the service environment; however, a complex algorithm is required to recognize features [13]. In addition, this method is difficult to apply to environments composed of monotonous structures such as corridors and factory walls having a small number of features.

To solve this problem, research is being actively conducted on estimating the position of a camera using artificial markers such as augmented reality (AR) or quick response (QR) markers [14,15,16,17,18]. Even though these methods use a low central processing unit (CPU), relatively fast and accurate position recognition is possible because artificial markers are recognized through their patterns even in a monotonous environment [14,19,20,21]. In addition, it is possible to evaluate the exact size of a marker by recognizing the four corners of the marker. Marker-based camera position recognition is used not only to recognize the position of a mobile robot but also to improve robot functions such as object position recognition, robot arm control, and human–robot interaction. However, the previous methods of estimating the position of a camera using markers experienced certain problems. First, the correct size and position of markers must be predefined. Therefore, this information must be known beforehand based on data or must be calculated using an additional algorithm. Furthermore, when an indoor environment changes or the driving region of the robot is expanded, a marker must be moved or added to a predefined, precise position. However, this becomes more difficult as the number of markers increases, and the region becomes wider. In addition, the data-based information in the position estimation algorithm must be updated when the size and position of a marker change. Second, when the position of the camera changes because of the movement of the robot, the distance required to recognize a marker decreases according to the size of the marker and the distance between the marker and camera. This limits the movement range of the mobile robot.

In this paper, a strategy for estimating the position of a camera mounted on a mobile robot is developed based on proposed markers. The proposed strategy includes three methods to overcome the abovementioned problems.

First, the proposed markers have information such as identification (ID), marker size, and marker type. Therefore, to recognize the position of the camera, information can be obtained directly from the marker. The advantage of this marker system is that a combination of markers of different size or having different information may be used without needing to update the internal parameters of the robot system. This is true even if the user frequently changes or adds to the identification information of the marker.

Second, the two markers, a nested marker and a hierarchical marker, are proposed to increase the recognition distance of the markers considering the movement of the camera due to the mobile robot. The nested marker is used when the camera moves vertically along the center of the marker like a drone. The hierarchical marker is used when the center of the marker and the height of the camera do not match such as when a wheeled robot moves horizontally on a floor. These markers contain marker size information based on internal patterns. 

Third, a new marker is added, or the position of an existing marker is changed, as the activity area of the robot changes or becomes wider. A method of predicting the position of the added or moved marker based on a known marker is proposed. Therefore, as the position of the marker is automatically updated, the process of accurately attaching the marker to the predefined position and updating the position information in the algorithm is not required.

Finally, the proposed methods are verified via experiments. First, the position of the camera is calculated for markers of various sizes using an existing method and the proposed method and then compared. The proposed method accurately estimates the camera position regardless of the marker size. Next, the method of calculating the position of the markers is verified using 12 markers attached at regular intervals along a corridor. Their positions are calculated using the proposed method and compared with the actual measured positions; the results show reasonable agreement. In addition, it was demonstrated that the location information of the markers is updated when the location of an existing marker is changed, or a new marker is added. Last, the recognition distances of three different markers and the proposed markers are compared. It was confirmed that the recognition distance for the proposed markers is more than that for the single-size markers.

## 2. Proposed Strategy for Camera Position Recognition

**Figure 2:**
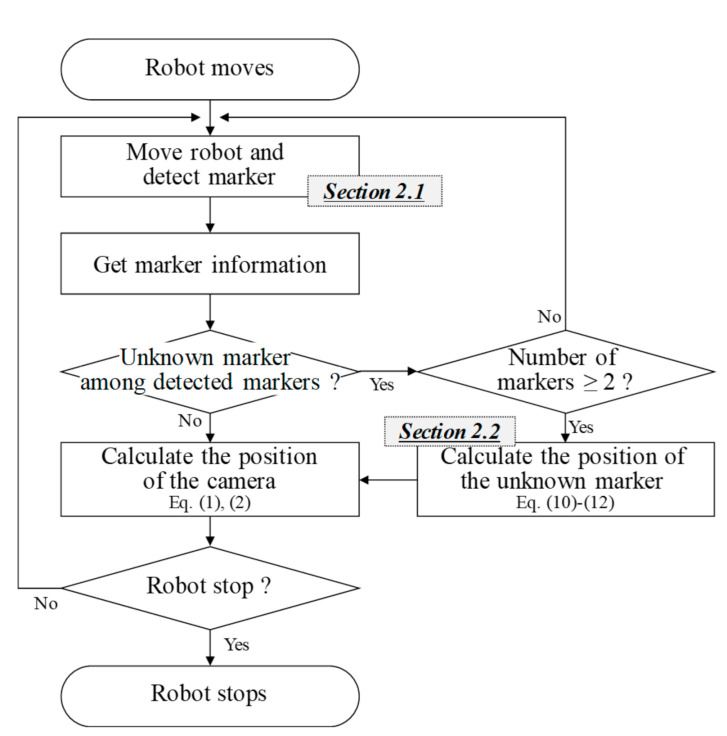
Flow chart of the proposed method.

This paper proposes a method for estimating the position of a camera based on markers containing size information. The proposed method considers a situation in which new markers are added and existing markers are repositioned as the driving area of the robot expands or the environment changes. Figure 2 shows the flow chart of the proposed method. 

**Figure 3:**
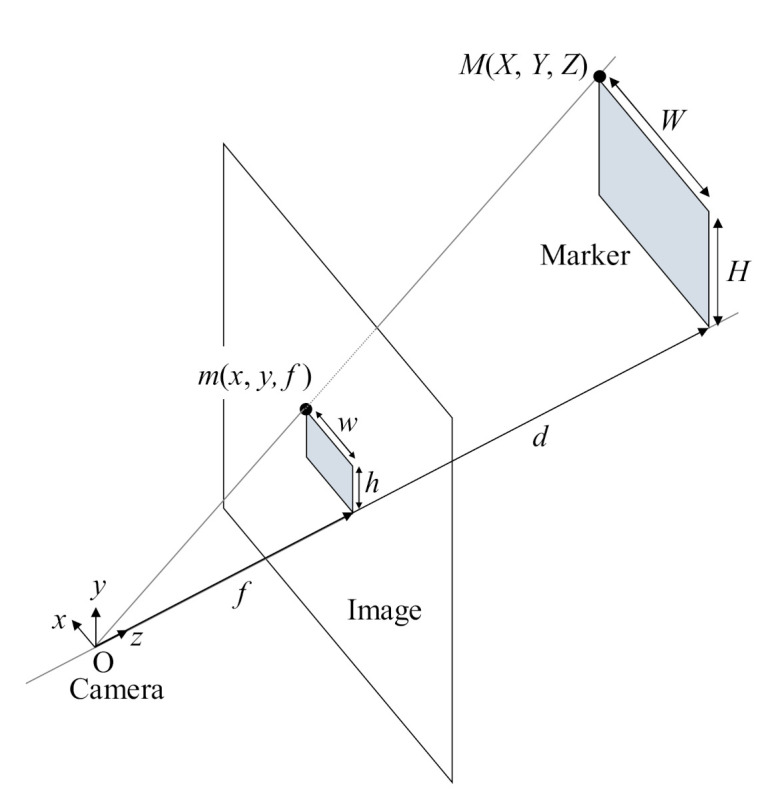
Relationship between camera, image, and marker.

First, as the robot moves, markers are detected through the camera. A region of interest (ROI) is set for recognizing markers within the image obtained using the camera. The marker image is obtained by extracting the corners of the marker in the ROI. This image is transferred to an information extraction module, and the ROI is moved if the marker is not recognized. Next, the information about the marker contained in the image is extracted. This information includes the type, length of one side, and identification (ID). The proposed marker has a unique ID because it consists of multiple markers for increasing the recognition distance of the camera. The relationship between a square marker and the camera position is derived using a mathematical method [22]. Figure 3 shows the relationship between the actual position (*M*) of the marker in three-dimensional (3-D) space, the image of the marker position (*m*) projected in two-dimensional (2-D) space, and the position of the camera (O) [22].

The coordinates of the marker in the projected image are expressed as follows:

(1) $$x=f\frac{X}{Z},y=f\frac{Y}{Z}$$

In addition, *d* is calculated using Equation (2).

(2) $$d=f\frac{w}{W}=f\frac{h}{H}$$

The distance between the camera and the marker is *d*; the focal length of the camera is *f*; the width and height of the actual marker are *W* and *H*, respectively; and the width and height of the marker in the projected image are *w* and *h*, respectively. 

The proposed marker is described in Section 2.1. When unknown markers without predefined location information are detected, their position is calculated. As the relative positions of the unknown markers are calculated based on the markers with predefined position information, the relative position can be calculated only if the number of recognized markers is more than two. A representative marker is selected from among the detected markers and the relative positions of the unknown markers are calculated based on the representative marker. Finally, the global positions of all markers are estimated using the global position of the representative marker and the calculated relative positions of the markers in a local coordinate system. The proposed method is based on the reference marker, and it assumes that there is no dead zone with no detected marker. The method for calculating the position of unknown markers is described in Section 2.2. After the position information of the detected markers is updated, the position of the camera is calculated using this information. First, the size of the markers in the image is calculated based on the resolution of the camera and the focal length of the camera for each pixel. Then, the camera position is evaluated using the calculated size of the marker in the image, the ID, and the actual size and type of the markers. If the marker information required for estimating the camera position is included inside a marker, it is possible to use a marker of the required size according to various environments without modifying the algorithm. In addition, it becomes easier to add and move markers when the driving area of the robot changes.

### 2.1. Proposed Markers Considering the Movement of the Camera

As the camera is mounted on the robot, the position of the camera continuously changes as the robot moves. Accordingly, the size of a marker in the projected image changes with the distance between the camera and marker. Table 1 shows the images of a marker with a size of 200 × 200 pixels and a marker with a size of 600 × 600 pixels captured by the camera at different distances from the markers. The first marker (200 × 200 pixels) is projected more clearly as the distance between the camera and marker decreases. However, the size of the marker in the image decreases as the distance increases. In contrast, the second marker (600 × 600 pixels) can be recognized even at a distance of 150 cm. However, only a part of the marker is visible at a distance of 10 cm, making it impossible to recognize.

This paper proposes a method that uses markers of different sizes to solve the abovementioned problem and increase the recognition distance of markers.

#### 2.1.1. Nested Marker 

**Figure 4:**
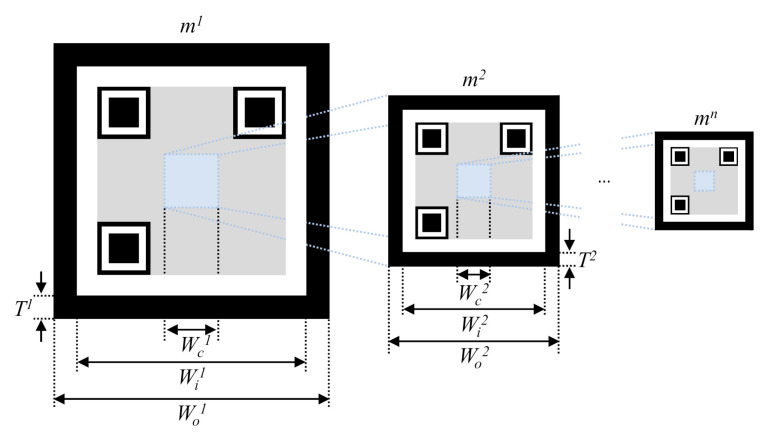
Structure of the proposed nested marker.

A nested marker is one of the markers applied to the proposed method for camera position recognition. Figure 4 shows the proposed nested marker. It is composed of *n* nested markers. All markers have a black border to make them easier to find within an image. The length and thickness of the *k*th marker are $W_{o}^{k}$ and *T^k^,* respectively. A white border is inserted within the black border to make the marker easier to recognize [23]. The thicknesses of the white and black borders are the same. Therefore, the length of one side of the white border ($W_{i}^{k}$) is calculated as follows:

(3) $$W_{i}^{k}=W_{o}^{k}-2T^{k}$$

As shown in Figure 4, lower markers are nested at the center of upper markers. The nested area excludes the area with the marker pattern, and the width ($W_{c}^{k}$) of the nested area is as expressed follows:

(4) $$W_{c}^{k}=\beta W_{o}^{k}$$

where *β* represents the ratio of the sizes of the overlapping upper and lower markers. 

*β* is set to be 20% or lower, and the total number of markers (*n*) is determined according to the recognition capability of the camera.

**Figure 5:**
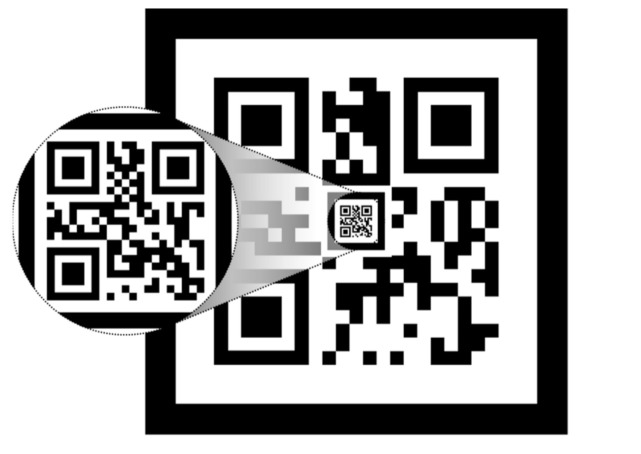
2-D nested marker.

Figure 5 shows a 2-D nested marker. Each marker includes information about its size.

As the proposed marker is nested based on the center of markers, the recognition distance of the marker can be increased. Therefore, the proposed nested marker is suitable for flying robots like drones that take off and land vertically. This is because the center of the marker can be matched with the camera facing the ground.

#### 2.1.2. Hierarchical Marker 

**Figure 6:**
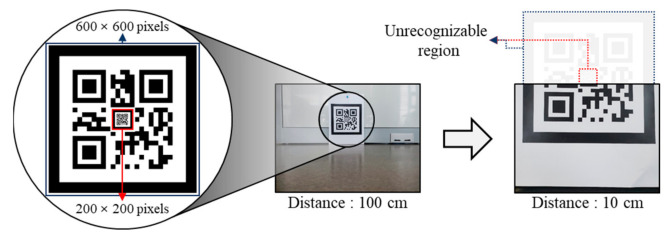
Problem in using nested marker with mobile robot.

When the camera is mounted on a wheeled mobile robot that moves horizontally with respect to the ground, the image captured by the camera consists of the lower end of the larger marker when the distance between the robot and marker is less. As a result, the markers nested inside the larger marker are not visible. Figure 6 illustrates this problem.

The Intel RealSense D435 camera is used. A marker with a size of 200 × 200 pixels is nested inside a marker with a size of 600 × 600 pixels. When the distance between the camera and the nested marker is 10 cm, the marker is not recognized because it is out of the measuring area of the camera.

**Figure 7:**
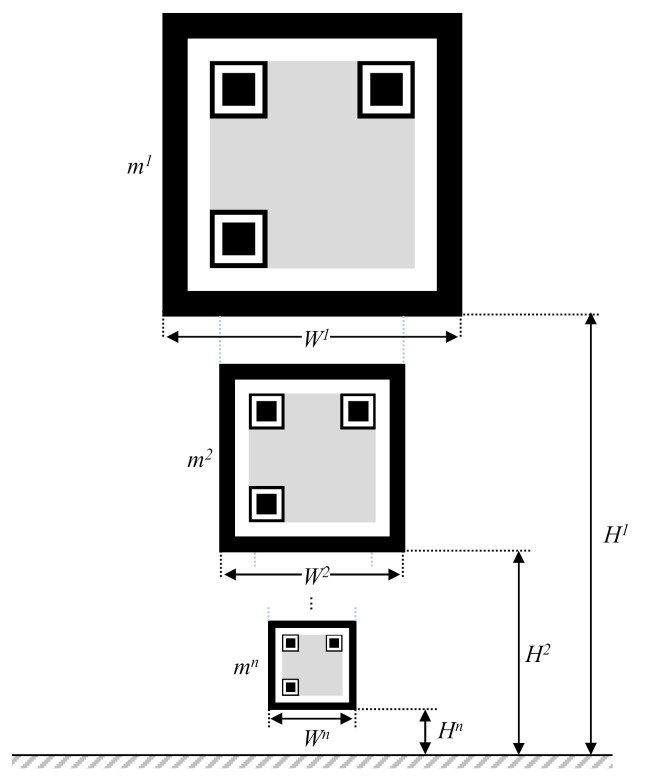
Structure of the proposed hierarchical marker.

This problem is related to the height and angle of view of the camera. A hierarchical marker is proposed to overcome this problem. Multiple markers are hierarchically stacked in this marker, as shown in Figure 7. The height and width of the *k*th marker is *H^k^* and *W^k^* respectively.

**Figure 8:**
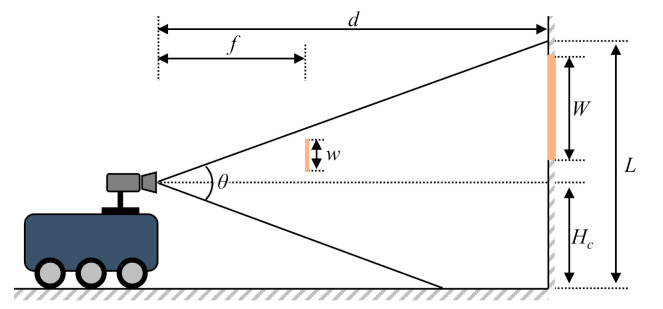
Relationship between the camera, the marker, and the marker image.

Figure 8 shows the relationship between the camera, marker, and marker image recognized by the camera. This relationship is used to derive the distance (*d*) from the center of the camera to the marker and the height (*L*) that the camera can measure, as follow:

(5) $$\frac{f}{d}=\frac{w}{W}$$

(6) $$d=f\frac{W}{w}$$

(7) $$L=d\tan\left(\frac{\theta }{2}\right)+H_{c}$$

where *f* is the focal length of the camera and *θ* is the maximum angle of view of the camera. 

These values are determined based on the specifications of the camera.

**Figure 9:**
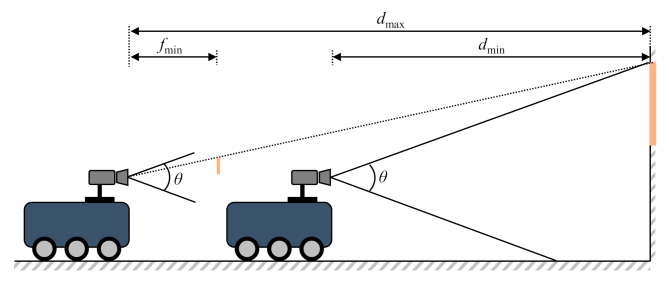
Maximum and minimum recognition distances.

The abovementioned equations are used to calculate the maximum and minimum distances for recognizing a single marker and are calculated as shown in Figure 9.

(8) $$d_{\max}=f_{\min}\frac{W}{w}$$

(9) $$d_{\min}=\frac{L-H_{c}}{\tan\left(\frac{\theta }{2}\right)}$$

where *f*_min_ is the minimum focal length of the camera.

Based on the previously calculated maximum and minimum recognition distances for a single marker, the condition for recognizing a marker in all sections as the robot moves is determined as follows:

(10) $$d_{\min}^{k}\leq d_{\max}^{k+1}$$

**Figure 10:**
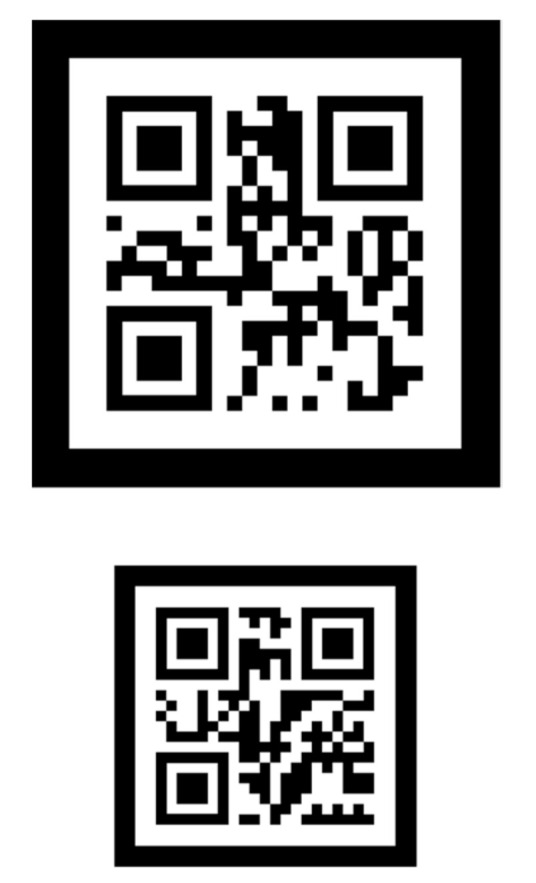
2-D hierarchical marker.

Figure 10 shows the proposed 2-D hierarchical marker.

#### 2.1.3. Characteristics of the Proposed Markers 

The proposed markers have information such as identification (ID), marker size, and marker type. Therefore, to recognize the position of the camera, the information of the marker can be obtained directly from it. The advantages of this marker system are as follows:

(1) A mixture of markers of different sizes or containing different included information may be used.
(2) It does not need to update the internal parameters of the robot system even if the user frequently changes or adds to the identification information of the marker.

Table 2 shows the characteristics of the existing markers used in the camera positioning system. The proposed markers can hold a larger amount of information than the others. This allows the markers containing more information to recognize the position of the camera. The robot system does not need to store this information, which allows it to have the advantage of adding markers at any time without changing the internal parameters of the robot system.

### 2.2. Estimating the Position of Unknown Markers 

This section explains the calculation of the position of a marker with unknown position information among detected markers. The proposed method determines the global positions of all markers by calculating the relative positions between them based on the markers defined in the global coordinate system. Markers are defined as follows:

- Reference marker: A marker whose position is predefined or calculated in the global coordinate system.
- Representative marker: A marker for calculating the relative positions of markers in the local coordinate system.
- Unknown marker: A marker whose position information is unknown because it has been newly added or moved.

**Figure 11:**
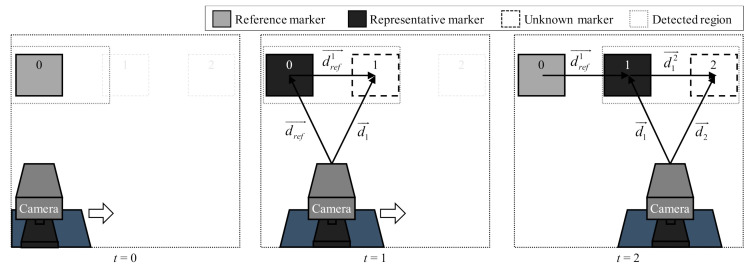
Process of calculating unknown marker position.

When two or more markers are recognized by the camera, the relative position of an unknown marker is calculated based on a representative marker. Among the recognized markers, a representative marker is determined and used to calculate a relative position vector. Figure 11 shows the process of calculating the marker position as the camera moves.

When *t* = 0, the camera recognizes only the reference marker. Therefore, no calculation is performed because there are fewer than two recognized markers. A new marker (marker 1) is recognized at *t* = 1, Then, a reference marker is determined as a representative marker to calculate the relative position of marker 1. In addition, the relative position between the two markers and the absolute position of marker 1 are calculated.

(11) $$\vec{d_{ref}^{1}}=\vec{d_{ref}}-\vec{d_{1}}$$

At *t* = 2, marker 1 is selected as a representative marker because its position information was calculated in the previous step. Then, the relative and absolute positions of marker 2 are calculated in a similar manner.

(12) $$\vec{d_{1}^{2}}=\vec{d_{1}}-\vec{d_{2}}$$

(13) $$\begin{array}{ll} \vec{d_{ref}^{2}} & =\vec{d_{ref}^{1}}+\vec{d_{1}^{2}}\\ & =\left(\vec{d_{ref}}-\vec{d_{1}}\right)+\left(\vec{d_{1}}-\vec{d_{2}}\right)\\ & =\vec{d_{ref}}-\vec{d_{2}} \end{array}$$

The position information of unknown markers is updated as this process is repeated.

This method can be used to predict the position of markers not only when markers are moved or added based on environmental changes but also when markers are added to inaccurate positions.

## 3. Verification of Proposed Method

### 3.1. Marker-Based Camera Position Recognition

**Figure 12:**
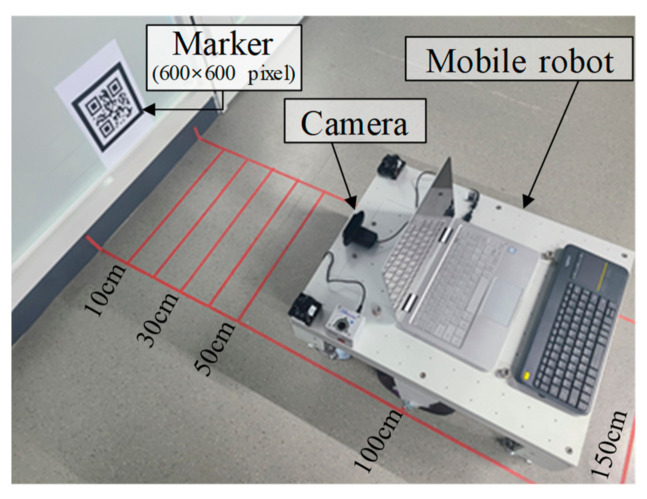
Test setup for verification of proposed method.

The proposed method for camera position recognition was verified by comparing it with a previous method using three single markers of different sizes. The sizes of the three markers were 200 × 200, 400 × 400, and 600 × 600 pixels, respectively, and an Intel RealSense D435 camera was used [23]. The distances between the markers and camera were calculated based on the Kanade–Lucas–Tomasi tracking algorithm of OpenCV [25]. The parameters for the previous method were set considering each pixel. As shown in Figure 12, the camera position was calculated at distance of 10, 30, 50, 100, and 150 cm.

Table 3 shows the camera positions calculated using the proposed and previous methods.

The camera position, calculated using the previous method, showed an error of at least 0.03% and at most 0.7% when the actual size of the marker matched the size of the marker set in the camera estimation algorithm. When the marker size was set as 200 × 200 pixels in the previous method, the error in estimating the camera position was small for the marker with a size of 200 × 200 pixels. However, it was calculated as 1/2 and 1/3 for the markers with sizes of 400 × 400 pixels and 600 × 600 pixels, respectively. On the contrary, as the proposed method acquired information from the markers and adaptively estimated the sizes of the markers, the error in the estimated camera position was 0.03% to 0.7% for all three markers.

### 3.2. Recognition Distance of the Proposed Markers

**Figure 13:**
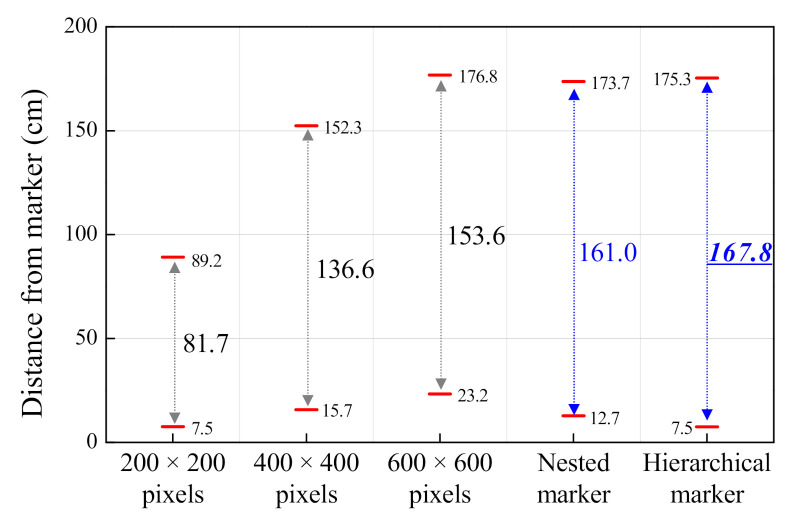
Recognition distances for different markers.

The effectiveness of the proposed nested marker and hierarchical marker was validated by comparing the measured recognition distances of the proposed markers and three conventional markers of different sizes. The nested marker was composed of two markers, one with a size of 600 × 600 pixels and the other with a size of 200 × 200 pixels. The hierarchical marker was constructed using markers with size of 200 × 200, 400 × 400, and 600 × 600 pixels. The camera was mounted on a mobile robot 30 cm above the ground. The marker was attached to a wall 50 cm above the ground. Figure 13 shows the test results.

It was verified that the recognition distance increased when two proposed markers were used as compared to when a single-size marker was used. In addition, the minimum recognition distances of the hierarchical and nested markers were 7.5 cm and 12.7 cm, respectively. The recognition distance of the hierarchical markers was approximately 58.7% less than that of the nested marker because, as mentioned in Section 2.1.2, the 200 × 200 pixels’ marker was not recognized as the camera was approaching it. Therefore, the hierarchical marker is suitable for maximizing the recognition distance in the case of a camera moving horizontally with respect to the ground, such as in a wheeled mobile robot.

### 3.3. Position Recognition for Unknown Marker

**Figure 14:**
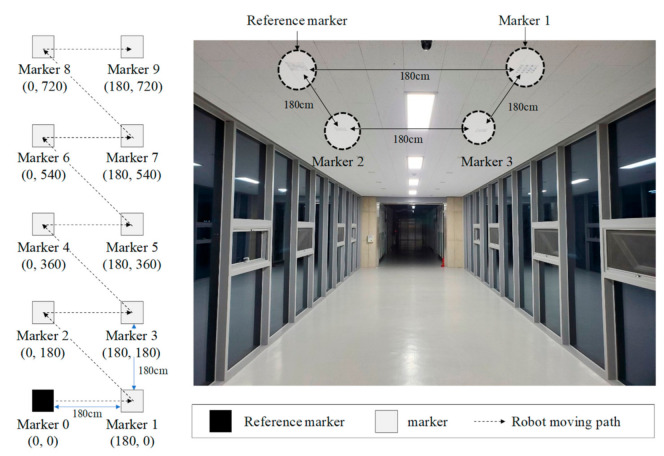
Test setup to verify method of calculating marker position.

Figure 14 shows the test setup used to verify the proposed method of calculating marker position [26]. Ten markers were attached to a corridor at equal intervals of 180 cm. The wheeled mobile robot moved from marker 0 to 9 at a speed of 0.1 m/s [27]. The global position of the reference marker (marker 0) was set as 0, 0. The positions of the markers were measured at 10 Hz.

Table 4 lists the global positions of the markers calculated using the proposed method and the actual positions of the markers. The results showed reasonable agreement.

**Figure 15:**
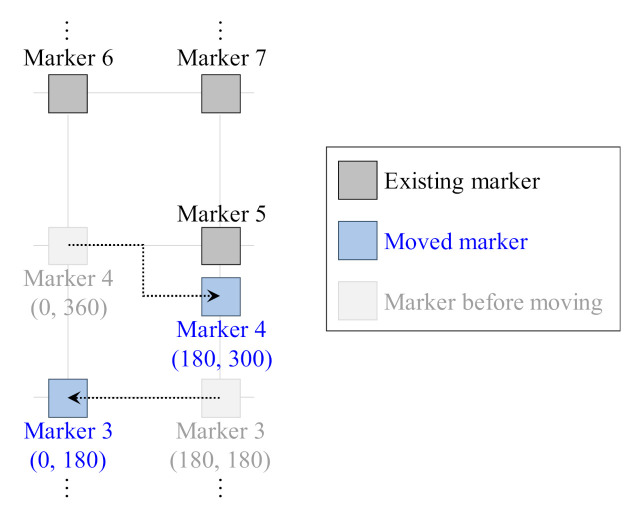
Test condition to verify method of calculating position of moved marker.

In addition, tests were performed to move markers and add new markers considering the frequent movement of markers or the use of additional markers owing to the expansion of the region. Figure 15 shows the test conditions for predicting marker movement. The position of marker 4 was changed from (0, 360) to (60, 420) in the global coordinate system, and the position of marker 5 was changed from (180, 300) to (180, 360). 

After changing their positions, the global positions of the markers were estimated. Table 5 shows the actual and estimated position of markers 3 and 4 before and after being moved. The global positions of the markers were estimated accurately even after they were moved. 

**Figure 16:**
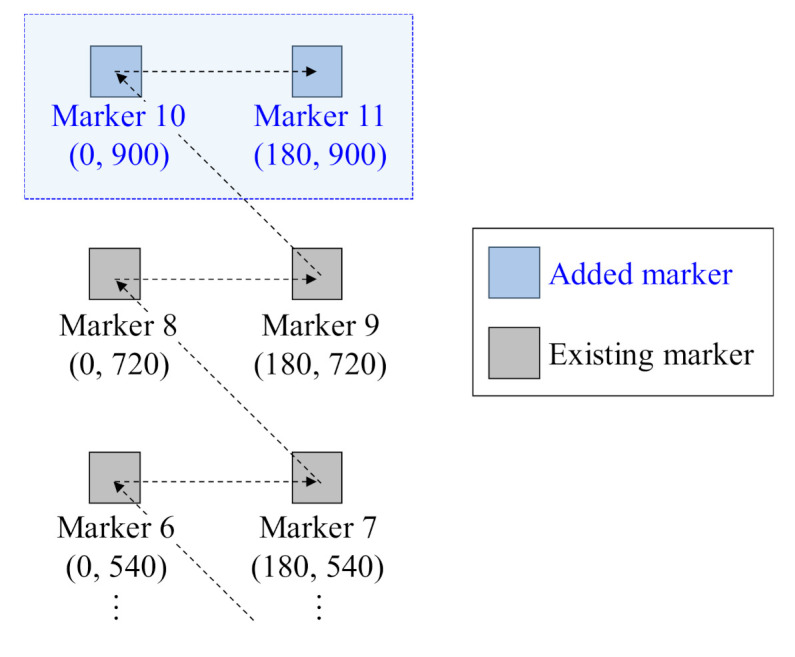
Test condition to verify method of calculating position of added marker.

Two new markers (markers 10 and 11) were attached to the corridor to confirm the recognition of newly added markers, as shown in Figure 16, and the global positions of the markers were estimated.

Table 6 shows the actual and estimated global positions of all markers after the new markers were attached and the robot moved along the same path. It was confirmed that the global positions of the existing markers were updated, and the global positions of the new markers were estimated using the proposed method.

## 4. Conclusions

This paper proposed a method for recognizing the position of a camera mounted on a mobile robot based on marker information while considering an increase in the area where the robot is driven or a change in the environment. The proposed method not only used novel markers to increase the recognition distance of the camera but also calculated the position of added and moved markers to respond to environmental changes. Two new markers were proposed depending on the driving pattern of a robot. In addition, a method was proposed for calculating the position of a marker with unknown position information based on reference marker. The proposed methods for camera position recognition were verified by evaluating the camera position and comparing it with the position determined using a previous method. The method of calculating the position of an unknown marker was verified by calculating the position of the marker using the proposed method and comparing it with the actual measured position. In addition, the positions of markers were estimated and updated based on the movement of the marker or the addition of markers. The recognition distances of the proposed markers were evaluated and compared with the recognition distances of the three conventional markers of different sizes. The results of the experiments confirmed the effectiveness of the proposed method.

In order to approach and apply more real-world environments, further studies are required to tackle the problem of marker recognition degradation because of changes in light and to increase the accuracy of marker recognition by applying probability filters.

## Figures and Tables

**Table 1:** Images of markers at different distances.

Distance (cm)	200 × 200 (pixels)	600 × 600 (pixels)
10	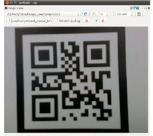	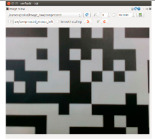
50	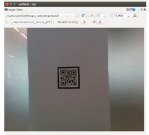	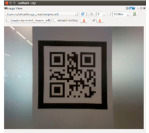
150	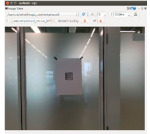	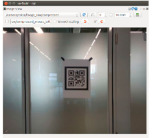

**Table 2:** Characteristics of markers used in camera positioning systems.

Marker	Size of Information within Marker (Information)	Storage Location of Marker Information		Parameter Change When Marker Size Is Changed
		Coordinate	Size	
AR	5 bytes (ID)	Robot system	Robot system	Need
QR	About 2953 bytes	Marker	Robot system	Need
ArUco [24]	5 bytes (ID)	Robot system	Robot system	Need
Proposed Marker	About 2953 bytes (ID, Size, Type)	Marker	Marker	Needless

**Table 3:** Position of camera.

Marker Size (pixel)	Distance (cm)	Previous Method (cm)			Proposed Method (cm)
		200	400	600	
200 × 200	10	10.3	20.7	31.4	10.3
	30	30.1	60.5	92.7	30.1
	50	50.3	100.6	154.1	50.0
	100	Not recognized			
	150				
400 × 400	10	Not recognized			
	30	15.0	30.0	45.9	30.0
	50	25.1	50.4	77.1	50.3
	100	50.1	100.4	153.3	100.4
	150	73.6	151.2	228.3	150.1
600 × 600	10	Not recognized			
	30	9.8	19.8	30.0	30.0
	50	16.4	32.8	50.1	50.1
	100	32.6	65.5	100.3	100.3
	150	49.2	98.3	150.2	150.2

**Table 4:** Positions of markers.

Marker Number	Actual Position (*x*, *y*)	Estimated Position (*x*, *y*)
0	(0, 0)	(0, 0)
1	(180, 0)	(182.9, −1.2)
2	(0, 180)	(0.47, 180.2)
3	(180, 180)	(182.5, 178.4)
4	(0, 360)	(0.91, 363.8)
5	(180, 360)	(183.4, 362.9)
6	(0, 540)	(2.3, 544.0)
7	(180, 540)	(185.0, 542.2)
8	(0, 720)	(3.1, 725.9)
9	(180, 720)	(180.8, 726.7)

**Table 5:** Position of the moved marker.

Marker Number	Actual Position (*x*, *y*)	Estimated Position (*x*, *y*)
3	(180, 180)	(180.4, 178.8)
	↓	
	(0, 180)	(0.0, 180.2)
4	(0, 360)	(0.9, 361.2)
	↓	
	(180, 300)	(182.3, 299.2)

**Table 6:** Positions of existing and added markers.

Marker Number		Actual Position (*x*, *y*)	Estimated Position (*x*, *y*)
0	Existing marker	(0, 0)	(0, 0)
1		(180, 0)	(181.8, 0.5)
2		(0, 180)	(1.17, 180.1)
3		(180, 180)	(180.4, 178.8)
4		(0, 360)	(0.93, 361.2)
5		(180, 360)	(180.3, 362.9)
6		(0, 540)	(2.6, 541.8)
7		(180, 540)	(182.2, 540.8)
8		(0, 720)	(3.5, 723.5)
9		(180, 720)	(182.1, 722.4)
10	Added marker	(0, 900)	(4.54, 903.9)
11		(180, 900)	(185.8, 903.3)

## Data Availability

Not applicable.
